# The hidden scabies: a rare case of atypical Norwegian scabies, case report and literature review

**DOI:** 10.1186/s13052-023-01547-z

**Published:** 2024-01-17

**Authors:** Angela Mauro, Cristiana Colonna, Silvia Taranto, Vittoria Garella, Francesca Castelletti, Laura Giordano, Nicola Adriano Monzani, Luca Bernardo

**Affiliations:** 1 Pediatric Rheumatology unit, Department of Childhood and Developmental Medicine, Fatebenefratelli-Sacco Hospital, Milan, Italy; 2 Pediatric Dermatology Unit, Department of Clinical Sciences and Community Health, Fondazione IRCCS Ca’ Granda Ospedale Maggiore Policlinico, Milan, Italy; 3 Pediatric Department, “Vittore Buzzi” Children’s hospital, Milan, Italy; 4 Pediatric Unit, Department of Childhood and Developmental Medicine, Fatebenefratelli-Sacco Hospital, Milan, Italy

**Keywords:** Scabies, Children, Norwegian scabies, Down syndrome, Immunodepression

## Abstract

**Background:**

Norwegian scabies is a rare dermatological manifestation that usually affects the most fragile populations, such as elderly and immunocompromised patients, and its diagnosis is quite complex, due to its low prevalence in the general population and because of a broad spectrum manifestation.

**Case Presentation:**

Here we describe a rare case of Norwegian scabies that was previously misdiagnosed in a sixteen year old patient affected by Down syndrome and we conducted a non-systematic literature review about this topic. Lesions were atypical, pruritic and associated with periodic desquamation of the palms and soles and after a series of specialist evaluations, she finally underwent topical treatment with complete remission.

**Conclusion:**

It is therefore crucial to take in consideration the relation between Down syndrome and community acquired crusted scabies, to enable preventative measures, early detection, and proper treatment.

**Supplementary Information:**

The online version contains supplementary material available at 10.1186/s13052-023-01547-z.

## Background

Scabies is a global infestation caused by the species-specific ectoparasitic mite *Sarcoptes scabiei* (var. hominis) [1]. Transmission is facilitated by close contact with an infested person and sharing living quarters [2]. The two major clinical variants of scabies are classic scabies and crusted scabies (Norwegian scabies). The most common, known as classic scabies, has a modest mite burden and it is characterized by diffuse intense itching, typically nocturnal, and numerous erythematous papules frequently excoriated. Nodules, which present as skin-colored, red-brown, or violaceous, may develop as a result of an exacerbated hypersensitive reaction as well as from rubbing and scratching. Lesions are normally symmetrical, commonly appearing on the fingers, wrists, elbows, axillae, areolae, peri-umbilical skin, waist, genitalia, knees, buttocks, and feet [3]. Intense skin infestation by *Sarcoptes scabiei*, as well as widespread crusty, hyperkeratosis papules, plaques, and nodules, are the hallmarks of crusty scabies, also known as Norwegian scabies [4, 5]. The atypical clinical aspects could make the diagnosis quite challenging [6]. Thus, it could be be strongly suspected upon the patient history and physical examination. A definitive diagnosis is achieved by direct observation with optical microscope of the mite, eggs, or faecal pellets. A negative preparation does not exclude the scabies, but in those cases, dermoscopic examination may be used as a helpful adjunctive diagnostic tool. Diagnosis, however, is often established by a positive response to treatment [3, 6].

Patients who are immunocompromised, particularly those receiving immunosuppressive medication or have cognitive impairment, are more likely to acquire crusty scabies. Although the correlation between ineffective immune system is used to explain the connection between Down syndrome and crusted scabies, this association is still not fully understood [5, 7].

We describe a case of Norwegian scabies in an adolescent patient with Down Syndrome, who underwent misdiagnosis with a significant delay in the treatment.

## Case presentation

**Fig. 1:**
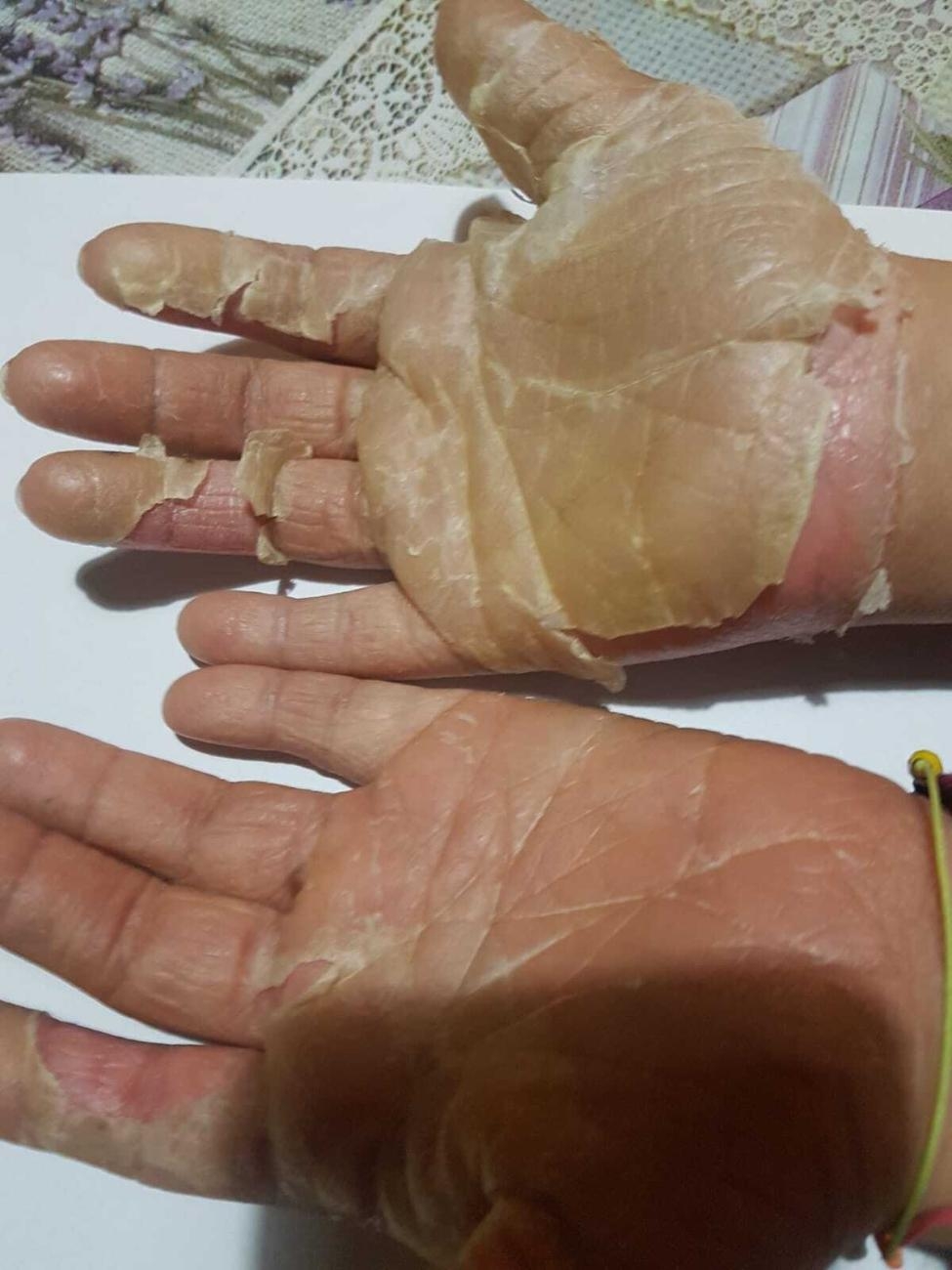
Desquamation of the soles of f palms

**Fig. 2:**
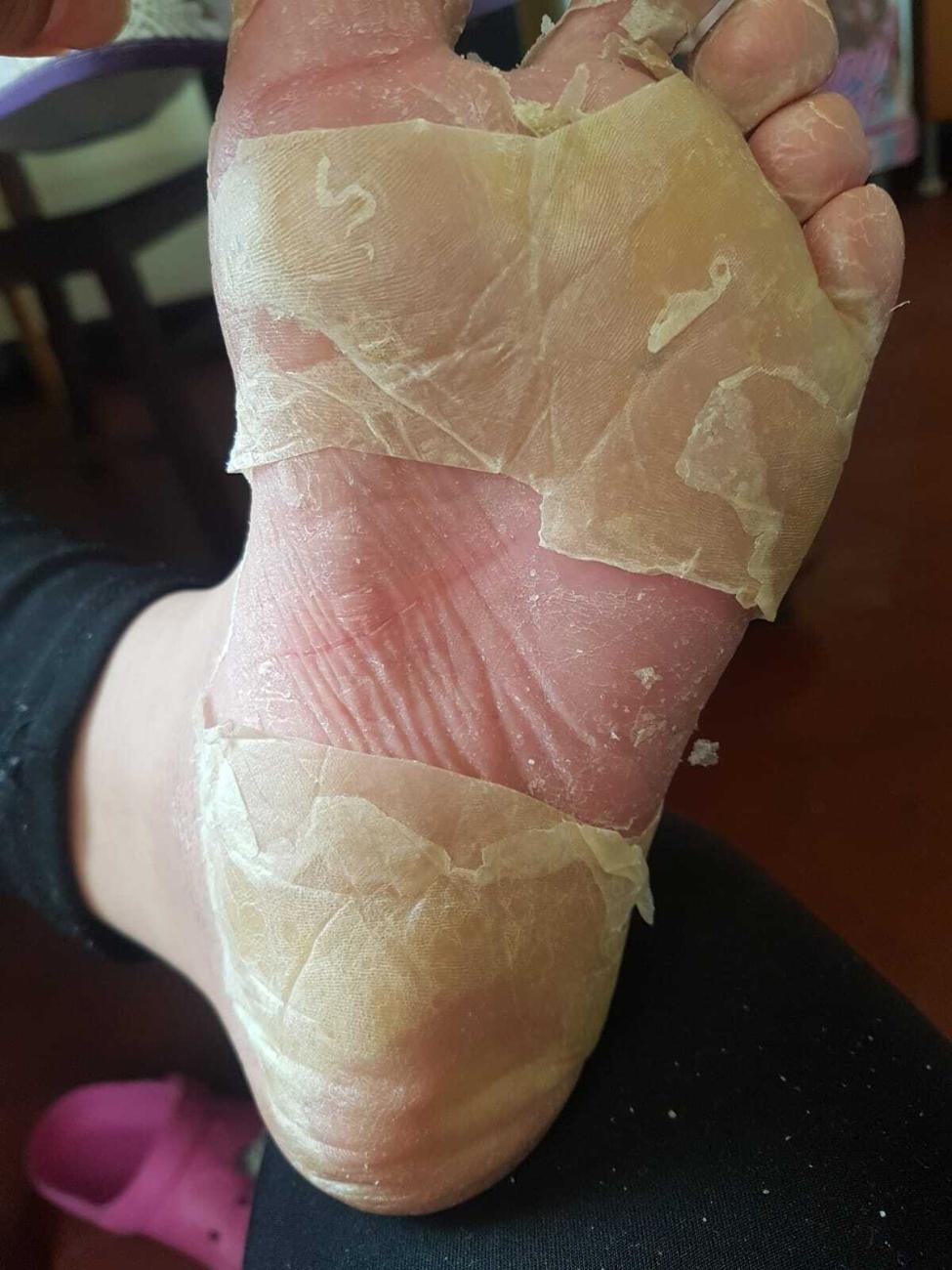
Desquamation of the soles of feet

**Fig. 3:**
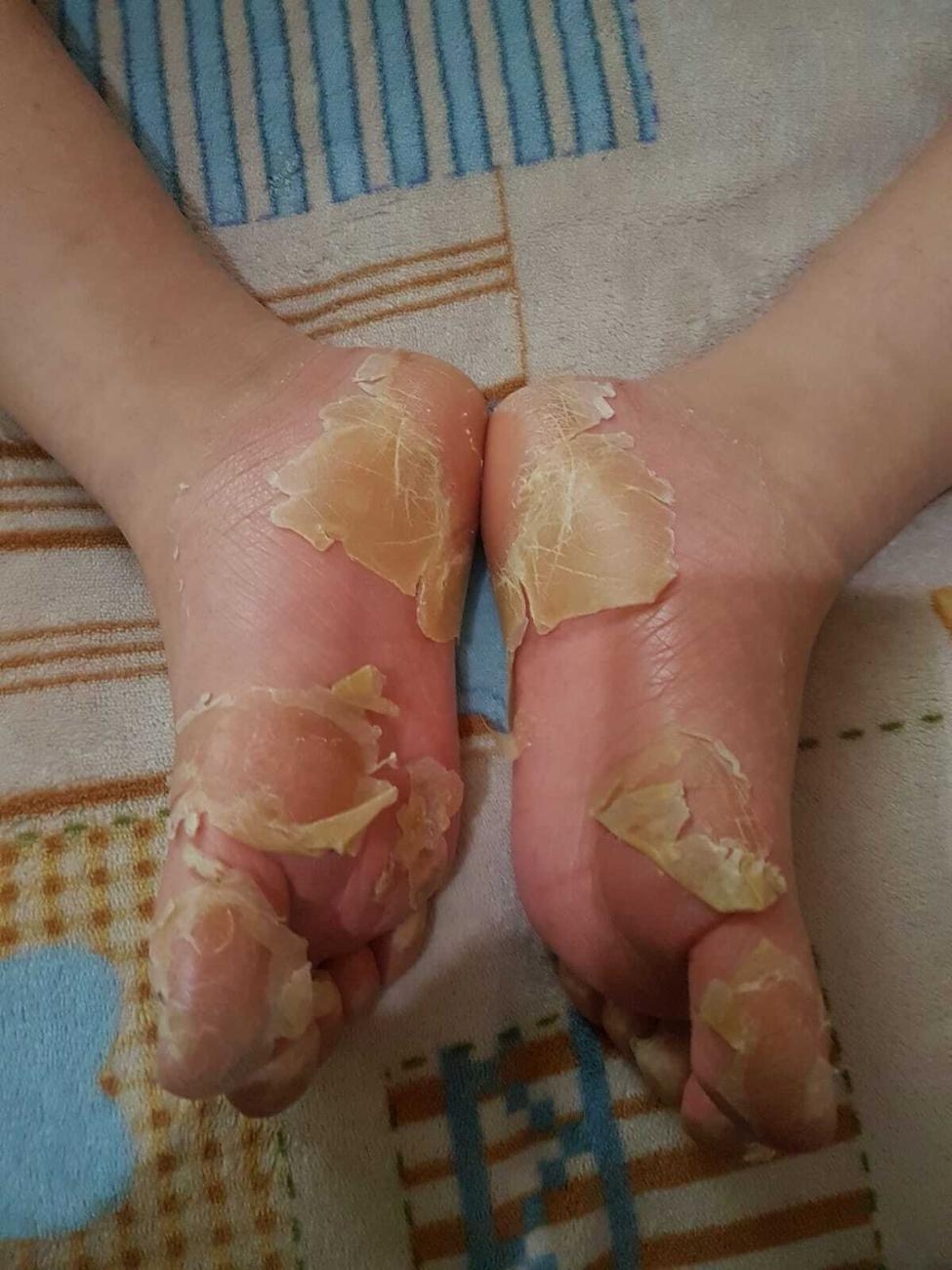
Desquamation of the soles of feet

A sixteen year old girl with Down syndrome, formerly 36 weeks of gestational age with previous congenital heart disease, was referred to our institution for widespread itching, worsening at night, and several episodes of desquamation occurring at the level of the soles of feet and palms (Figs. 1, 2 and 3).

Symptoms began 3 months earlier, in concomitant to rhinitis and intermittent fever. She was evaluated at the Paediatrics’s Emergency Department and tested positive for Streptococcus Pyogenes oropharyngeal swab, therefore, she was discharged with a diagnosis of pharynges-tonsillitis and she was prescribed oral antibiotic and antihistamine therapy.

During the same period, her cohabiting sister was diagnosed with scabies, thus the whole family was treated with 5% permethrin cream, with consequent improvement of symptoms. Unfortunately, none of the family members underwent post-treatment control evaluation.

Two months later, because of the persistence of the lesions, the patient was re-evaluated by a dermatologist who described the presence of partially exfoliated, indurated and erythematous skin on soles of feet and palms with large flakes desquamation, with areas of hyperpigmented papular crusted skin on the trunk and limbs. Taking in consideration her past medical history, the suspicion of Kawasaki Disease or scarlet fever arose with no laboratory confirmation. After several Paediatric and Dermatological evaluations and laboratory testing including an echocardiogram, whose findings were consistent with the previous findings, she was referred to our institution for a Paediatric Rheumatologic evaluation, which suggested the hospitalization to our Paediatric Ward (see Supplementary Timeline). During the hospital stay, in-depth blood tests were performed, demonstrating a slightly increased ESR and serum amyloid A, positive ANA (1:160) and a reduction of LyB with a relatively higher level of LyT CD8 compared to LyT CD4 (Table 1). To complete the diagnostic assessment, an allergological and ocular assessment were performed too, excluding any condition related to rheumatologic, topical allergies and signs of uveitis.

**Table 1:** Patient blood exams during hospitalization to our facility

Laboratory item	Value	Reference value
Hemoglobin	143	125–155 g/L
White Blood Cells	6.43	4.5–10 × 10^9^/L
Absolute neutrophils	3.99	1.91–6.23 × 10^9^/L
CD3+	82.81% (1.532)	64–83% (1.1–2.4 × 10^3^/µL)
CD4 + CD3+	35.32% (0.66)	34–60% (0.6–1.5 × 10^3^/µL)
CD8 + CD3+	43.8% (0.818)	13–35% (0.22–0.87 × 10^3^/µL)
CD19+	3.37% (0.062)	6–19% (0.14–0.5 × 10^3^/µL)
CD4/CD8	0.81	
Platelets	303	169–359 × 10^9^/L
Blood smear	Ipochromic and presence of anysocytosis of RBCs. Few target RBCs.	
Immunoglobulin M	0.93	0.4–2.3 g/L
Immunoglobulin G	22.16	7–16 g/L
Immunoglobulin A	1.97	0.7 -4 g/L
Immunoglobulin E	5.91	< 100 g/L
Tetanus IgG	0.12	< 0.05–0.1 no protection 0.11–0.5 protection present, booster recommended
C3	1.49	0.9–1.8 g/L
C4	0.21	0.1–0.4 g/L
Amyloid A	12.3	< 8 mg/L
ANA	1:160 (dotted)	< 1:80
ENA	0.2	< 0.7

RBC: Red Blood Cells; ANA: Anti-Nuclear Antibodies; ENA Extractable Nuclear Antigens

She also underwent a dermatologic evaluation in a paediatric dermatological referral centre, during which moderate erythema was evident on the soles and palms, skin was partially infiltrated and there were scales with large flaps on the palmar-plantar margins. The back of the hands and feet were thickened with mild erythema and frankly eczematous appearance. There were three papular crusted lesions at pubis, left thigh root, left groin, as well as there were pigmented areas at the margins of the axillary cavities, suggestive of previous scabies nodules. Her mother also reported she was suffering from itching for 3–4 days, especially at the level of her wrists and hands. Scales were obtain from the papular crusted lesions of the groin and from the interdigital papular areas of the adolescent. As well as, the mother’s skin scales from hands were evaluated under the light microscope, observing ectoparasitic excrements, therefore defining the diagnosis of Norwegian scabies. All family started treatment with Topical Benzil Benzoate 25% once a day for 3 days and then for another 3 days after a 5-day break. During the 5-day break she performed treatment with topic Desoximetasone once a day for 5 days, with brilliant response. At 1 month follow-up she was in good condition without any signs of previous infection.

Written informed consent to use of patient’s de-identified, anonymized, aggregated data and their case details was obtained for publication.

## Discussion and conclusion

In our case report we describe an unusual presentation of Norwegian scabies and its implication in the delay of diagnosis and misdiagnosis.

Norwegian scabies is typically associated with systemic immunosuppression conditions (HIV or lymphoproliferative disorders, post-transplant patients and those on long-term corticosteroids), severe systemic illness (e.g. autoimmune disease) or neurological disease. The association between crusted scabies and immunosuppression is widely known and observed, because of the inadequate host’s response to the mite proliferation [8]. The link between this severe form of scabies and neurocognitive disorders and developmental disabilities, specifically Down syndrome, despite being well documented in the literature, is still poorly defined. Various hypotheses have been proposed as explanatory mechanisms, the most plausible is related to the connection between cognitive delay and the diminished ability to interpret and express pruritus [9, 10], but also to subtle abnormalities of the immune system which are observed in this population [10]. It is known that Down Syndrome is a primary immunodeficiency disorder characterized by a significant decrease in switched memory B cells and T cells, both of CD4 + and CD8 + cells [11]. Also, CD8 + T cells are known to be crucial skin-infiltrating cells, therefore the combination of the imbalance in skin-homing cytotoxic T cells and low B cell count, may result in the worsening of crusted scabies skin lesions [4]. It is interesting to observe that in our case the immune pattern of the patient was exactly the one described in the literature.

To compare our case with other similar cases reported in the literature, we conducted a narrative review searching on PubMed and Embase using the following key-words: ‘down syndrome’, ‘trisomy 21’, ‘crusted scabies’, ‘norwegian scabies’, ‘children’ and ‘pediatric’. We selected all case reports about children aged between 1 and 18 years with Down syndrome and crusted scabies (Table 2). We found 8 case reports matching the aforementioned criteria, characterized by age ranging between 11 months and 14 years old (mean age 7,3 years old) and a female predominance (6 F, 1 M, 1 not reported) [9, 12–18]. In 6 of them the disease presented with itching and in half of them one or more family members were affected by scabies too. None of them was affected by specific comorbidities other than congenital cardiomyopathy and atopic dermatitis that fall within the framework of the underlying disease. Analysing all the selected cases, 5 out of 8 were diagnosed through direct microscopy of skin scrapings, while the others were diagnosed clinically, dermoscopically or in one case histologically. Moreover, it is important to point out the significant time interval from the onset of symptoms and the diagnosis (ranging from 2 to 18 months, with a mean delay of 7 months). This diagnostic delay, that has shown to be systematic in the literature of scabies may strongly worsen the quality of life of patients’ as in the case of our patient, who suffered from chronic severe episodes of desquamation and daily itching [13, 18]. Finally, 5 of the 8 aforementioned cases were treated with a combination of oral and topical treatment, while the others solved with topical treatment [9, 13–15, 18].

**Table 2:** Cases of Norwegian Scabies in children with Down Syndrome reported in literature

	Age	Other Diseases	Gender	Family symptoms	Itching	Diagnosis	Time to diagnosis	Therapy
**Our case**	16 yy	Congenital cardiomyopathy	F	+	++	Microscopy of skin scrapings	3 mm	Topical
**Cebeci et al.** [12]	28 mm	Atopic dermatitis	F	+	++	Dermoscopic examination	4 mm	Topical
**Lee et al.** [9]	11 yy	Atopic dermatitis	F			Microscopy of skin scrapings	NR	Topical + oral
**Murugaiyan et al.** [13]	11 yy	NR	M	+	++	Microscopy of skin scrapings	18 mm	Topical + oral
**Mantero et al.** [14]	14 yy	Congenital cardiomyopathy			+	Clinical	2 mm	Topical + oral
**Franco et al.** [15]	9 yy	NR	F	NR	++	Biopsy	6 mm	Topical + oral
**Thean et al.** [16]	11 mm	Malnutrition	F	+	NR	Microscopy of skin scrapings	NR	Topical
**Assaf et al.** [17]	8 yy	NR	F	+	++	Microscopy of skin scrapings	6 mm	NR
**Fonseca et al.** [18]	3 yy	NR	F	NR	++	Microscopy of skin scrapings	7 mm	Topical + oral

F: Female; M: Male; NR: Not Reported

The anti-scabies treatment has to be carried out appropriately, both in the patient and her contacts. In order to successfully treat scabies and to prevent its spread, it is important to identify the factors that can influence treatment outcome. Some common risk factors are incorrect application of permethrin, reinfection because of the partial treatment of contacts and resistance of mites toward permethrin [19]. Furthermore, impaired cognitive function was identified as a factor related with treatment failure [20]. A less effective treatment with hyperkeratotic skin must be considered as risk factor [19].

In conclusion, despite being a widely known disease, crusted scabies may still be misdiagnosed, especially in more fragile populations, as in our case. The interplay between prevention, use of diagnostic tools and proper treatment still remains a crucial strategy in the management of crusted scabies in this population.

### Electronic supplementary material

Below is the link to the electronic supplementary material.

Supplementary Material 1

## Data Availability

Data sharing is not applicable to this article as no datasets were generated.
