# Supplementary material for: The hidden scabies: a rare case of atypical Norwegian scabies, case report and literature review
Source: Ital J Pediatr. 2024 Jan 17;50:7. doi: 10.1186/s13052-023-01547-z (PMC10795281; doi:10.1186/s13052-023-01547-z)
Supplement: Supplementary file 1 — Supplementary Material 1 [file 13052_2023_1547_MOESM1_ESM.pdf]

Dates	Relevant Past Medical History and Interventions
	Down syndrome, formerly 36 weeks of gestational age, previous congenital heart disease. February 2023 rhinitis and intermittent fever with a positive test for Streptococcus Pyogenes oropharyngeal swab, therefore treated with oral antibiotic and antihistamine therapy. February 2023 treatement with 5% permethrin cream because her cohabiting sister was diagnosed with scabies, without post-treatment control evaluation.

Date	Summaries from Initial and Follow-up Visits	Interventions	Results
April 2023	Hospitalization to Fatebenefratelli's Paediatric Ward	Blood tests<br>Allergological and ocular evaluations	Slightly increased ESR and serum amyloid A, positive ANA (1:160) and a reduction of LyB with a relatively higher level of LyT CD8 compared to LyT CD4.<br>Exclusion of any condition related to rheumatologic, topical allergies and signs of uveitis.
April 2023	Dermatologic evaluation	Light microscope	Diagnosis of Norwegian scabies
April 2023	Dermatologic evaluation	Treatment	Topical Benzil Benzoate 25% once a day for 3 days and then for another 3 days after a 5-day break. During the 5-day break topic Desoximetasone once a day for 5 days.
May 2023	1 month's dermatologic follow-up	Dermatologic evaluation	Disease resolution
